# Computational modeling with forward and reverse engineering links signaling network and genomic regulatory responses: NF-κB signaling-induced gene expression responses in inflammation

**DOI:** 10.1186/1471-2105-11-308

**Published:** 2010-06-08

**Authors:** Shih Chi Peng, David Shan Hill Wong, Kai Che Tung, Yan Yu Chen, Chun Cheih Chao, Chien Hua Peng, Yung Jen Chuang, Chuan Yi Tang

**Affiliations:** 1 Department of Computer Science, National Tsing Hua University, Hsinchu, 30013, Taiwan, ROC; 2 Department of Chemical Engineering, National Tsing Hua University, Hsinchu, 30013, Taiwan, ROC; 3 Institute of Bioinformatics and Structural Biology, National Tsing Hua University, Hsinchu, 30013, Taiwan, ROC; 4 Department of Resource Center for Clinical Research, Chang Gung Memorial Hospital, Taoyuan, 333, Taiwan, ROC

## Abstract

**Background:**

Signal transduction is the major mechanism through which cells transmit external stimuli to evoke intracellular biochemical responses. Diverse cellular stimuli create a wide variety of transcription factor activities through signal transduction pathways, resulting in different gene expression patterns. Understanding the relationship between external stimuli and the corresponding cellular responses, as well as the subsequent effects on downstream genes, is a major challenge in systems biology. Thus, a systematic approach is needed to integrate experimental data and theoretical hypotheses to identify the physiological consequences of environmental stimuli.

**Results:**

We proposed a systematic approach that combines forward and reverse engineering to link the signal transduction cascade with the gene responses. To demonstrate the feasibility of our strategy, we focused on linking the NF-κB signaling pathway with the inflammatory gene regulatory responses because NF-κB has long been recognized to play a crucial role in inflammation. We first utilized forward engineering (Hybrid Functional Petri Nets) to construct the NF-κB signaling pathway and reverse engineering (Network Components Analysis) to build a gene regulatory network (GRN). Then, we demonstrated that the corresponding IKK profiles can be identified in the GRN and are consistent with the experimental validation of the IKK kinase assay. We found that the time-lapse gene expression of several cytokines and chemokines (TNF-α, IL-1, IL-6, CXCL1, CXCL2 and CCL3) is concordant with the NF-κB activity profile, and these genes have stronger influence strength within the GRN. Such regulatory effects have highlighted the crucial roles of NF-κB signaling in the acute inflammatory response and enhance our understanding of the systemic inflammatory response syndrome.

**Conclusion:**

We successfully identified and distinguished the corresponding signaling profiles among three microarray datasets with different stimuli strengths. In our model, the crucial genes of the NF-κB regulatory network were also identified to reflect the biological consequences of inflammation. With the experimental validation, our strategy is thus an effective solution to decipher cross-talk effects when attempting to integrate new kinetic parameters from other signal transduction pathways. The strategy also provides new insight for systems biology modeling to link any signal transduction pathways with the responses of downstream genes of interest.

## Background

Signal transduction is a complex process in which a cell converts environmental signals to a series of intracellular biochemical reactions. Diverse cellular stimuli can create a wide variety of transcription factor activities through signal transduction pathways, resulting in differential gene expression that dictates subsequent cellular behaviors. Although a great deal of effort has been made in modeling signal transduction pathways or gene regulatory networks independently, a strategy to link the signaling pathway with downstream gene expression responses seems to be lacking. Thus, a systematic approach is needed to integrate experimental data and theoretical hypotheses, to identify the physiological consequences of environmental stimuli.

Over the past few years, a considerable number of studies have reported various systematic modeling protocols for the reconstruction of large-scale cellular signaling networks [1-8]. Besides the qualitative analysis of the signaling networks, mathematical approaches for the quantitative modeling and simulation of signal transduction pathways have also been developed [9-15]. Most of these quantitative signaling models are kinetic reactions represented by the assemblage of ordinary differential equations (ODE) [16]. The ODEs are designed to simulate dynamic changes throughout continuous but limited time points that define a function of rate changes for one independent variable and one or more of its derivatives with respect to that variable. Given the determined kinetic parameters, such ODE models provide a "forward-engineering" framework to simulate the spatiotemporal dynamics of the system. The time profiles of the target transcription factor activation in response to various stimuli can be obtained by this established approach.

In gene regulatory network modeling, some wet-bench experimental approaches have been used to detect the gene expression and transcriptional activities. Microarray technology is a powerful high-throughput technique enabling biologists to simultaneously measure the expression profile of tens of thousands of genes under prescribed conditions [17,18]. As for transcriptional activities, the electrophoretic mobility shift assay (EMSA) [19,20] is an affinity electrophoresis technique that can determine single protein or protein-DNA complex binding activity at a time. However, the ability to broadly assess the activities of transcription factors is still much limited. Therefore, most computational transcription activity and gene regulatory network are reconstructed from genome wide gene expression data.

Such a data-driven approach to gene expression analysis provides systematic information of underlying gene regulatory systems and offers the possibility to infer the dynamics and mechanisms of transcription control by reverse engineering [21-28]. There are two kinds of reverse engineering strategies for modeling gene regulatory networks based on DNA microarray data, namely the "influence" and "physical" approaches [29]. The "influence approach" produces the genetic network that illustrates regulatory influences between RNA transcripts. This strategy can integrate information pertaining to the relationships between regulated genes, protein-protein interactions, and enzyme catalysis to establish network based on transcript profiling data when the expression of certain transcripts is highly correlated. However, influence models are difficult to interpret in the context of location and modification in the cell. The second strategy, known as the "physical approach", seeks to construct a physical interaction model between transcription factors and gene promoters. The transcriptional activities can be often predicted from gene expression data by a sigmoid function [30]. Moreover, factor analysis is another methodology to construct a physical regulation model which is represented as bipartite graph with transcription factors in the first layer and regulated genes in the second layer for reducing the dimensionality of the reverse engineering problem. In previous studies, principal component analysis (PCA) [31], independent component analysis (ICA) [32], and network component analysis (NCA) [33] have been applied to reconstruct transcription factor activities using gene expression profiles. PCA and ICA are traditional dimensionality reduction technologies. The transcription factor activity reconstructed by PCA and ICA is constrained, respectively, to be mutually orthogonal and statistical independent. These statistical assumptions do not match the real biological systems. However, NCA contrasts with traditional PCA and ICA in that it does not make any aforementioned statistical assumptions.

NF-κB is a transcription factor that has long been recognized as the "master switch" in regulating the expression of various cytokines and host response effectors, as well as a wide array of genes to control inflammation, cell survival, apoptosis, and immune defense responses [34]. NF-κB signaling can be initiated from membrane receptors, such as Toll-like receptors, tumor necrosis factor alpha (TNF-α) receptors, and interleukin-1 (IL-1) receptors, either individually or synergistically. In the past few decades, many studies have tried to resolve the complex NF-κB dependent protein-protein and DNA-protein interactions, and significant progress has since been made on modeling signal transduction pathways and gene regulatory networks of the inflammatory response based on both biochemical and microarray data [35-40]. However, a systemic and dynamic view of how external stimuli evoke NF-κB-dependent signal transduction activities to the downstream gene expression still remained unclear.

To overcome this challenge, we proposed a new computational modeling approach by connecting transcription activities derived from the reverse engineering of gene expression profiling to the transcription activities simulated from a forward engineering signaling model, using the NF-κB signaling pathway with the corresponding gene responses as the case study. In this work, the NCA model was applied to reconstruct the regulatory activity of NF-κB using gene expression profile data obtained in response to specific external stimuli [41]. A kinetic model was used to simulate the IKK-NF-κB signaling pathway [42]. By mapping the NF-κB profiles generated from the reverse engineering gene expression profiling data and the ones from the forward simulation, the bridging IKK activities induced by external stimuli were inferred. Features of this inferred signaling process were then confirmed by independent experiments using similar stimuli. This strategy successfully linked the initial signaling pathway with the relevant gene regulatory network. It also successfully inferred and distinguished the corresponding stimuli from gene responses under different inflammation conditions. Taken together, the strategy discussed in the present study can help enhance our understanding of inflammatory responses during the infection process; it is also applicable to other cellular processes.

## Results and Discussion

**Figure 1:**
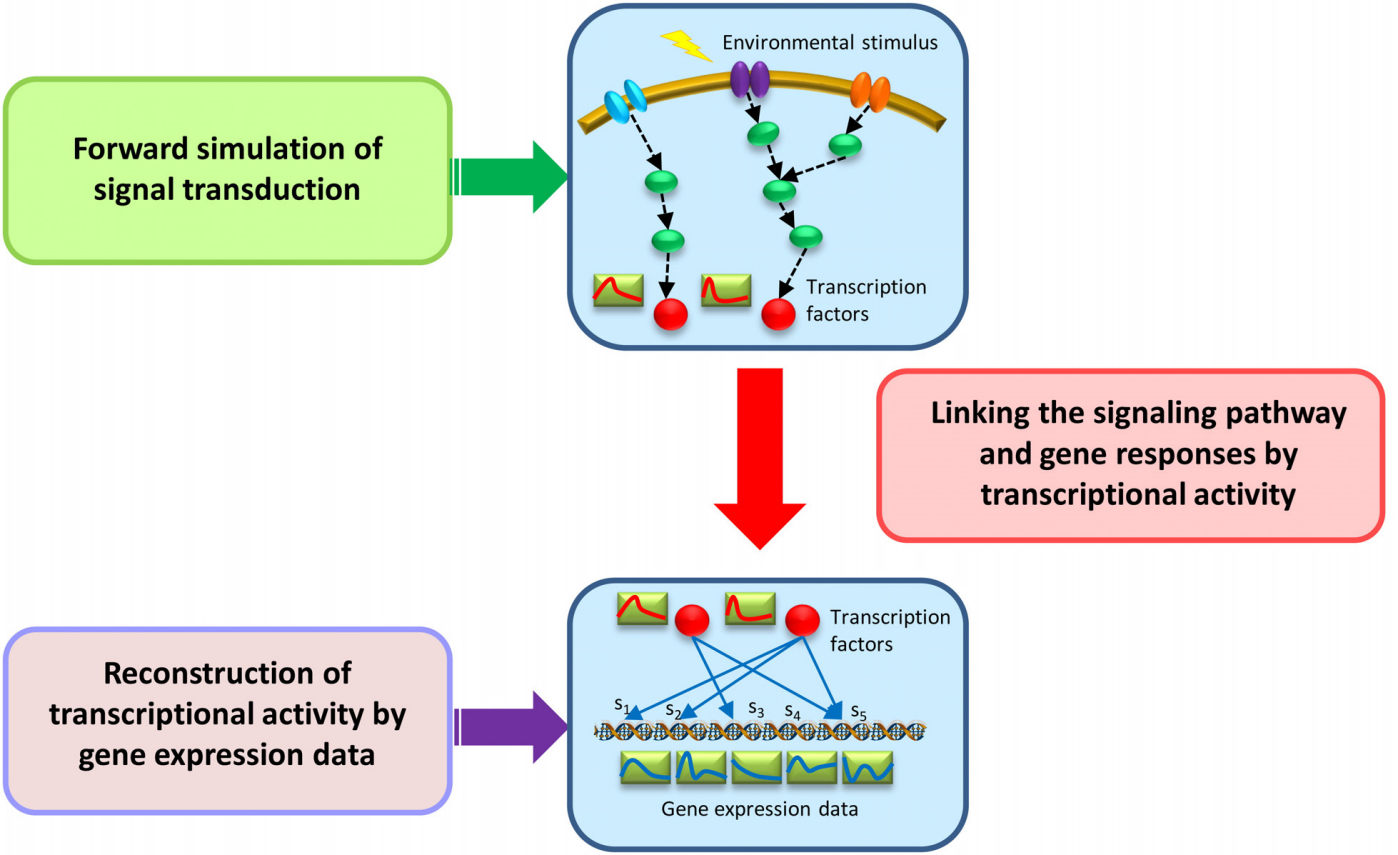
**The illustration of the overall strategy**. There are three parts of the strategy for linking signal transduction with gene responses. First, a reverse engineering model is used to reconstruct the regulators' activity profiles and relative regulatory strength from temporal gene expression data. Then the forward simulation model that describes the signal transduction pathway is constructed for simulating the regulatory profiles of the transcription factors. Finally, the forward-simulated transcription factor profiles are used to match with the activity patterns of the transcription factors that were obtained from the reverse engineering.

The novel approach presented in this study combines forward engineering and reverse engineering strategies to infer the relationship between an external signal and its resulting genomic responses within a cell. This novel strategy can be divided into three parts, as illustrated in Figure 1. The first two parts are used to produce the regulatory profile of the transcription factor from the signal transduction simulation and the subsequent gene response, respectively. Using temporal gene expression profiles and known transcriptional regulation relationships, a reverse engineering approach is used to reconstruct the activity profiles of transcription factors with the relative influence strength of the regulatory relationships, such as the binding affinity, between transcription factors and their target genes in the regulatory network. Then, the forward simulation model, which describes the signal transduction from the external stimulus to the specific transcription factor, is constructed for simulating the regulatory profiles of transcription factors that are associated with different stimuli. Finally, the forward-simulated transcription factor profiles are used to match with the dynamic activity pattern of transcription factors that are obtained from the reverse engineering. In the following subsections, we demonstrated an example that links the NF-κB signaling pathway with its consequential gene responses under different inflammatory stimuli. Thus, we demonstrated the feasibility and strength of this integrative approach to reconcile genetic responses induced by different stimuli into a gene regulatory model.

### The reconstruction of the transcriptional activity of NF-κB by NCA

The Network Component Analysis [33] is a natural model with a reverse-engineering approach that has been used to deduce transcription factor activities and regulatory control strengths using temporal gene expression data and transcriptional regulatory relationships. The NCA framework regards the gene regulatory network as a bipartite network model involving transcription factors and their target genes. The details of the NCA model are described in the Methods.

Lipopolysaccharide (LPS) is a kind of bacterial endotoxin widely used to stimulate inflammatory responses *in vitro*. The gene expression datasets in which cell cultures were treated with different doses of LPS from Boldrick et al. [41] were used. Briefly, Boldrick et al. added LPS to human peripheral blood mononuclear cells (PBMCs) at concentrations of 0.01 μg/ml, 0.1 μg/ml, and 1 μg/ml. The transcript profiles of the PBMCs were determined by microarray and evaluated before the infection and at 0.5, 2, 4, 6, and 12 hours after infection.

**Figure 2:**
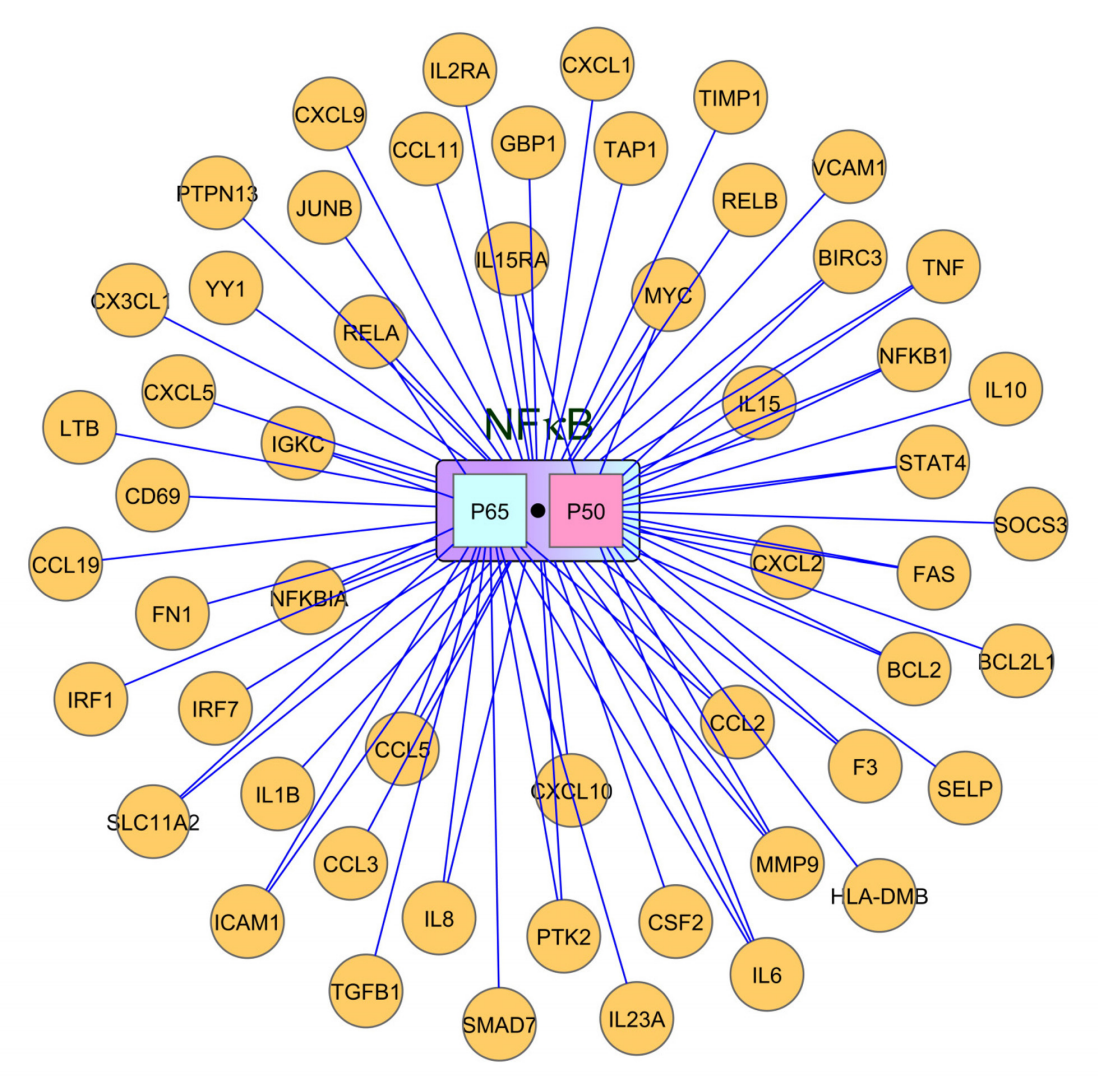
**The target gene regulatory network, NF-κB/RelA:P50**. The most common NF-κB in the canonical NF-κB signaling pathway is a heterodimer that is composed of RelA and P50. RelA and P50 also can form homodimers and regulate gene expression. The regulatory relationships of NF-κB/RelA:P50 were obtained from the TRANFAC database and the MetaCore™ analytical suite, which both are knowledge bases, and the data were collected from manually curated peer-reviewed literature. Based on these databases, we filtered out some target genes of NF-κB/RelA:P50 that expressed insignificantly in the LPS-induced gene profiles. The regulatory network of NF-κB/RelA:P50 finally consisted of 54 total target genes with 77 transcription regulatory interactions.

The transcriptional regulatory module of NF-κB was obtained from the TRANSFAC database [43] and MetaCore™ analytical suite (GeneGo Inc., St. Joseph, MI, USA). Both the TRANSFAC and MetaCore™ provide knowledge-based information, and all of the deposited data were collected from manually curated peer-reviewed literature. The TRANSFAC database contains expertly curated data on transcription factors, their experimentally proven binding sites, and regulated genes. MetaCore™ is an integrated database and software suite for pathway analyses of experimental data and gene annotation. Because this study was specifically aimed at simulating NF-κB activity, which represents a combination of dimerized proteins and cannot easily be quantitatively determined by experimental approaches, we hence focused on the primary transcriptional mediator, NF-κB/RelA-p50, of the canonical NF-κB signaling pathway only. RelA and p50 are the major and most common constituents of the NF-κB dimer complex in most cell types [44]. Either RelA or p50 can also form homodimers to regulate gene expression. Therefore, based on the TRANFAC and MetaCore™ data, a transcription regulatory network of NF-κB/RelA-p50 consisting of a total of 87 target genes with 125 direct transcription regulatory relationships was generated. However, the target genes of the NF-κB/RelA-p50 transcription factor that were not expressed significantly in the LPS-induced gene profiles were filtered. Finally, as shown in Figure 2, the regulatory network was reduced to 54 target genes, with 77 transcription-regulatory interactions. As expected, most of these genes, including IL-1β, IL6, IL8, TNF, CXCL1, and CXCL2, are related to pro-inflammatory functions.

**Figure 3:**
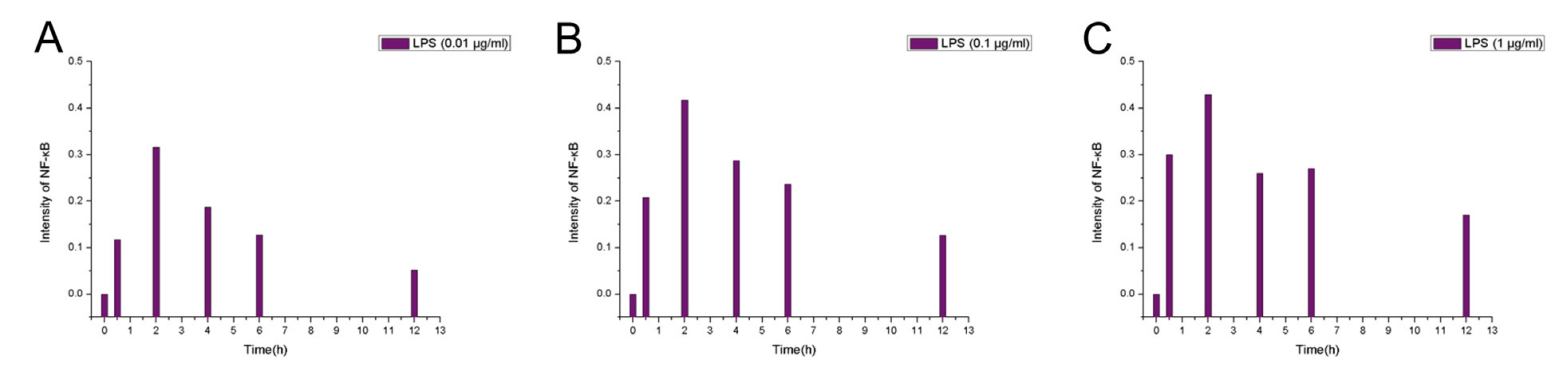
**The activity profiles of NF-κB reconstructed by NCA**. These profiles represent the dose-dependent NF-κB activity with relative activation strengths that were reconstructed by reverse engineering modeling framework (NCA) using gene expression data among three different doses of LPS stimuli. Each profile contains data points at 0, 0.5, 2, 4, 6, and 12 hours. (A) The activity profile of NF-κB with 0.01 μg/ml LPS. This profile has only one peak at 2 hours and then decreases, but the activity decreases more slowly after four hours. (B) The activity profile of NF-κB with 0.1 μg/ml LPS. This profile has only one peak at 2 hours and then decreases, but the activity decreases more slowly after four hours. (C) The activity profile of NF-κB with 1 μg/ml LPS. This profile peaks at 2 hours but has a period of sustained (non-decreasing) activity between 4 hours and 6 hours.

From this processed dataset, the NCA approach decomposes the gene expression matrix [E] into an influence-strength matrix [S] and a transcription factor activities matrix [A]. In order to reconstruct the dose-dependant transcription factor activity profiles with the relative activation strength among different doses of LPS stimuli, we merged the three gene expression matrices with the LPS doses of 0.01 μg/ml, 0.1 μg/ml, and 1 μg/ml into an expanded gene expressions matrix. Figure 3 shows the NCA-derived transcriptional activity profiles of NF-κB under different dose of LPS (The activity and strength matrices is available as Additional file 1). All three of the NF-κB profiles have an initial peak at two hours, but each NF-κB activity pattern has a different activation intensity. The difference in activation intensities is due to the activation intensity depending on the strength of the different LPS stimuli. This activity pattern coincides with the experimentally validated result demonstrating that the NF-κB activity peaks at approximately one to two hours after the induction by LPS [45].

It should be pointed out that even though the LPS dose changes over two orders of magnitude, the heights of the initial peaks of the reconstructed NF-κB activities differ by less than 50%. On the other hand, the increasing LPS dose produced profiles with different features, as described below during the later stage after two hours. Figure 3 illustrates that the activation intensities of all the three NF-κB profiles decrease after the peak at two hours. However, after four hours, the NF-κB activity decreases more slowly (Figure 3A and 3B) in the conditions of both of the lower doses of LPS (0.01 μg/ml, 0.1 μg/ml), whereas NF-κB maintains its activation intensity and results in a shoulder shape (Figure 3C) at six hours under the high dose of LPS (1 μg/ml). These patterns of reconstructed NF-κB activity were used in identification of corresponding IKK activity profiles.

### IKK-NF-κB signaling simulation

The nuclear transcription factor NF-κB is a protein complex that regulates numerous physiological processes [44,46] and plays a pivotal role in regulating the innate and adaptive immune responses [47-49]. Inactive NF-κB is retained in the cytoplasm while bound to an inhibitory factor, IκB. Upon stimulation by TNF-α, bacterial LPS or IL-1, NF-κB can be released from IκB and activated through a series of signal transduction pathways. Among these pathways, IKK proteins are the converging key regulators of NF-κB signaling. For example, when bacterial pathogens present virulent factors into host cells, IκB is phosphorylated by activated IKK and subsequently degraded by the ubiquitin-proteasome complex [50]. Upon IκB degradation, the activated NF-κB is then translocated into the nucleus where it regulates transcription from a large number of genes encoding components of the immune system, including a wide range of pro-inflammatory cytokines, chemokines, adhesion molecules, and inducible enzymes [44]. Accordingly, a multitude of signals transmitted from various receptors in the cell results in IKK activation, thus initiating the IKK-IκB-NF-κB signaling cascade that regulates NF-κB activity.

Previously, a computational model describing the inflammatory signal transduction dynamics in mammals from IKK to NF-κB has been proposed and experimentally validated by Hoffmann and colleagues [42,45,51,52]. Their model allows temporal simulations of the dynamic profile of NF-κB in the nucleus upon in the input of different profiles of IKK activity. In this study, we adapted the Hoffmann model [45,51,52] and expressed it in ODEs representing the mass balances of 24 components involving 72 reactions. The Hybrid Functional Petri-Net (HFPN) [53,54] was then used to carry out simulations of the kinetic model. HFPN uses uncomplicated graph annotations to define kinetic functions. The architecture of the model are shown in the supplementary data, and a detailed explanation is provided in the Materials and Methods section.

**Figure 4:**
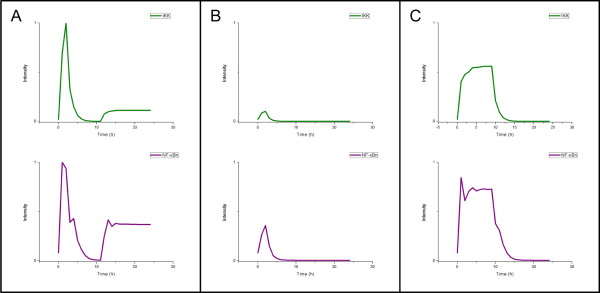
**Three examples of NF-κB signaling pathway simulation**. The NF-κB profile can be obtained by using various IKK profiles with different intensities and stimulation durations in the IKK-NF-κB signaling simulator. Three examples of IKK stimuli and the corresponding NF-κB profiles are shown. (A) The acute stimulus of IKK that has a peak at around two hours and a positive feedback control after ten hours. (B) A weak stimulus of IKK. (C) The IKK stimulus is not an impulse stimulation. It rises steeply during the first two hours and then increases slowly until ten hours.

The final outputs of the forward simulation model are the specific downstream transcription factor activity profiles, such as the NF-κB activity profile in this model. Figures 4A to 4C show three examples of NF-κB profiles generated from IKK profiles with different intensities and activation durations. In all cases, the corresponding NF-κB profiles demonstrated high correlations with the IKK profiles, with small time delay. In Figure 4A, the intensity of the NF-κB activity also peaked with an acute pulse of IKK at approximately two hours. Moreover, as the IKK activity increased again after ten hours, the intensity of NF-κB activity also increased. Figure 4B shows that a smaller increase of IKK activity will produce weaker NF-κB activity. Figure 4C represents another situation in which the IKK activation intensity persists for several hours and then drops off. The corresponding NF-κB activity also lasted for almost the same duration.

Based on this simulation model of IKK-NF-κB signaling, we can infer that IKK activity arises from the LPS stimuli by mapping the simulated NF-κB profiles to those derived from the gene expression datasets. Moreover, because the simulated IKK profiles have consistent activity with the derived NF-κB profiles, we would expect that the inferred IKK profiles would have similar features to the reconstructed NF-κB profiles from the gene expression data.

### Linking the NF-κB signaling pathway to the corresponding changes in gene expression

The computational model of the forward simulation for the NF-κB signaling pathway provides NF-κB profiles under different IKK stimuli. Given the reconstructed NF-κB profiles from the gene expression data and the computational model of the forward simulation under different IKK stimuli, we can generate IKK activity curves by matching the NCA-derived NF-κB profiles and the forward simulated NF-κB profiles initially. Then, the IKK activity curve that corresponds to the best-matched forward-simulated NF-κB profile will be selected.

**Figure 5:**
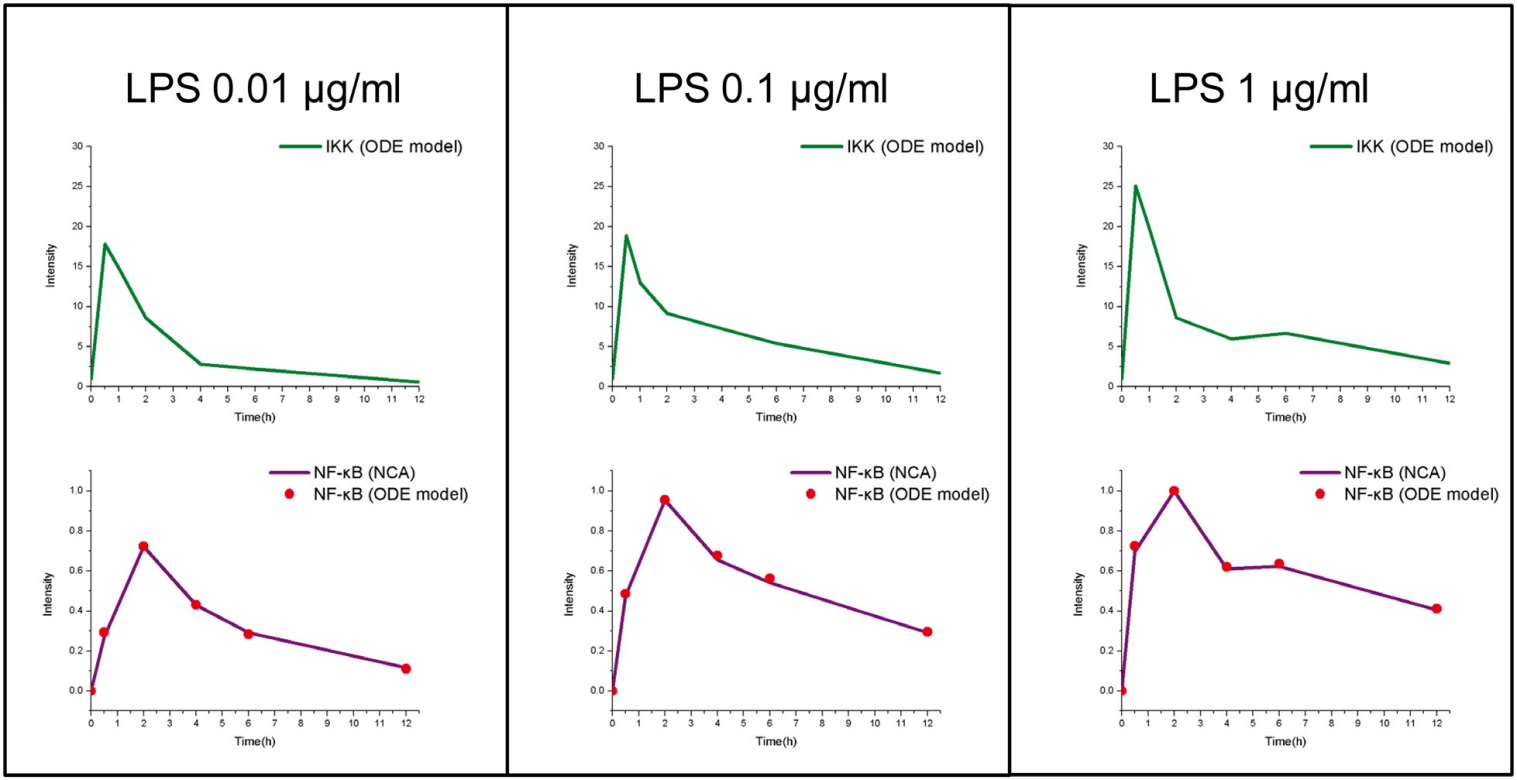
**Linking the NF-κB signaling pathway and gene responses by mapping the pattern of NF-κB profiles**. By simulating the NF-κB signaling pathway, one of the NF-κB profiles (pink data points) that is induced by a specified IKK profile (green line) matches the pattern of the NF-κB profile (purple line) reconstructed from the gene expression data produced by NCA among the three different doses of LPS stimulus. In each conditions of LPS stimulus strength, the pattern of the two NF-κB profiles can be mapped at the time points of 0, 0.5, 2, 4, 6, and 12 hours with a Pearson correlation of ~0.99.

Figure 5 shows, at the specified time points (0, 0.5, 2, 4, 6, and 12 hours), the IKK profiles that can produce the simulated NF-κB activity profiles best matched with those reconstructed from the gene expression by NCA. The patterns of two matched NF-κB profiles are highly correlated, with Pearson correlation coefficients of approximately 0.99. As expected, the IKK profile retains the key features of the NF-κB profiles: initial peaks were observed within half an hour of the stimuli; the amplitude of the initial peak increased slightly with order-of-magnitude changes in the extracellular stimuli. It is worth noting that a second peak and signals are found for the highest dose at six hours. To demonstrate the predictive power of our integrative model, these candidates of the IKK activity profile were further validated by an experimental IKK Kinase Assay carried out under the same experimental conditions of the reported gene expression datasets, with stimuli of three different LPS doses.

### Validations and model interpretation

**Figure 6:**
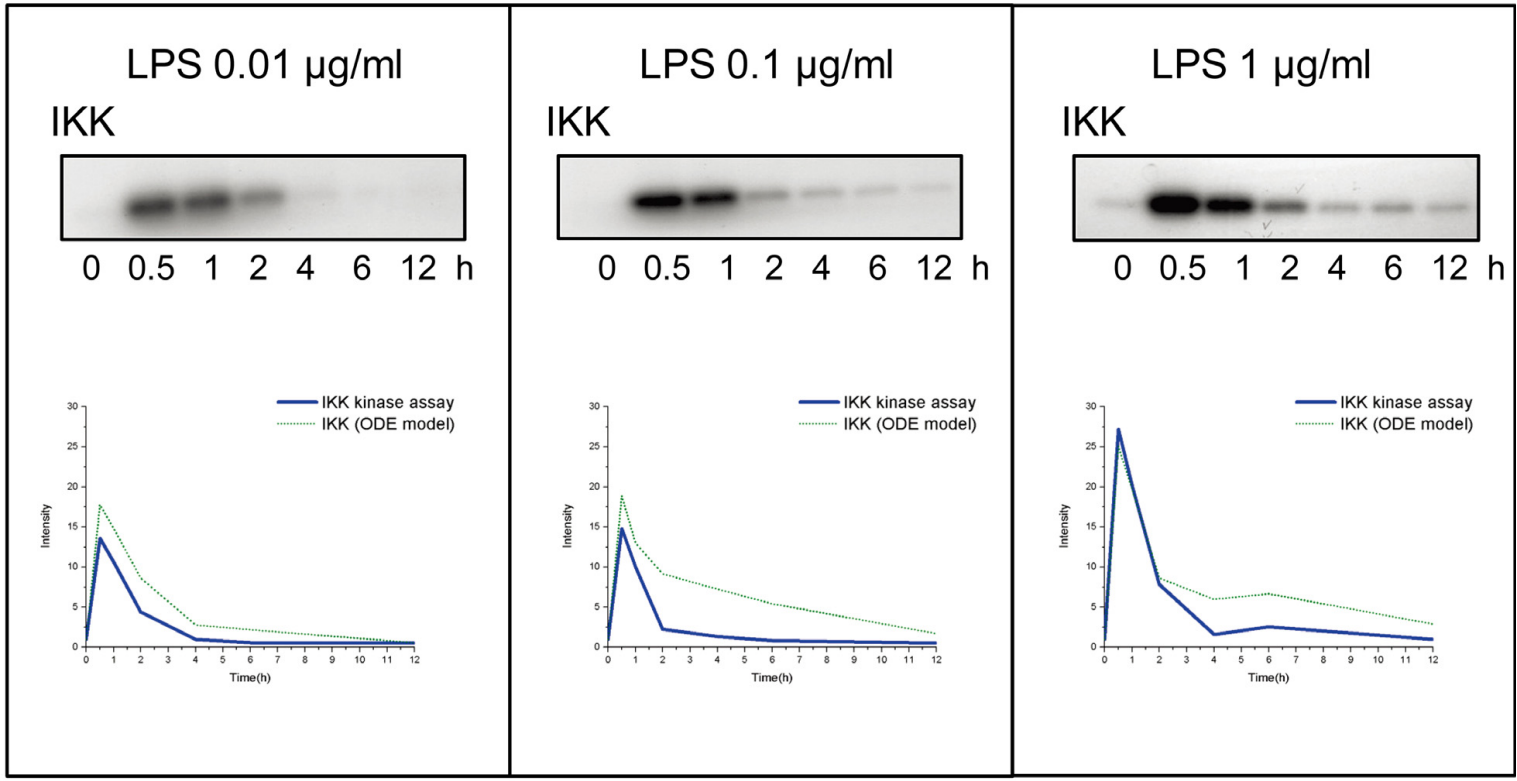
**The experimental validation of the IKK Kinase Assay**. The experimental validation of the IKK Kinase Assay under three different doses of LPS at the time points of 0, 0.5, 1, 2, 4, 6, and 12 hours. The blue lines are obtained by Image Q (GE Healthcare) that represents the western blot results of IKK activity. The green dotted lines represent the simulation results from forward engineering modeling framework (ODE model). The patterns of IKK activity from both the IKK Kinase Assay and the computational results are consistence. The ten-fold and hundred-fold dose of LPS appears not to trigger an extremely intense cellular response but induce periods of slow decay and non-decreasing inflammatory response between 4 to 6 hours respectively.

In order to validate the computational results, the IKK Kinase Assay was performed individually with LPS concentrations of 0.01 μg/ml, 0.1 μg/ml, and 1 μg/ml. Figure 6 indicates that the results of the IKK Kinase Assay are consistent with our IKK activity patterns derived from the computational prediction. Under the different stimuli strengths, all of the IKK profiles reached their peaks at 0.5 hours. Although there are ten-fold or hundred-fold differences in the LPS dose, the activity intensity of the IKK response did not show an increase of the same magnitude. The disparity between the stimulation intensity and the response activity is probably due to the limited number of receptors on the cell membrane or some damping mechanism of the signal transduction pathway. However, we observed that with the 1 μg/ml LPS stimulus, the IKK profile had a second wave at six hours. It is possible that large amounts of LPS cannot be completely consumed in the first wave of cellular reactions. Moreover, this disparity is also likely caused by positive feedback control the TNF-α and IL-1 autocrine signaling, to regulate cell proliferation and augment NF-κB activation [55,56]. Therefore, according to our model, the high dose of LPS appears not to trigger an extremely intense cellular response, but rather to induce a secondary reaction of an inflammatory response. This result is consistent with the hypothesis [57] that the innate response is not sufficient for a large number of invading microorganisms in an acute severe infection, which then results in a rapid multiplication of the microorganisms and induces the second response of the host. The circulating cytokines, such as TNF-α and IL-1, are thus produced overwhelmingly after some delay.

**Figure 7:**
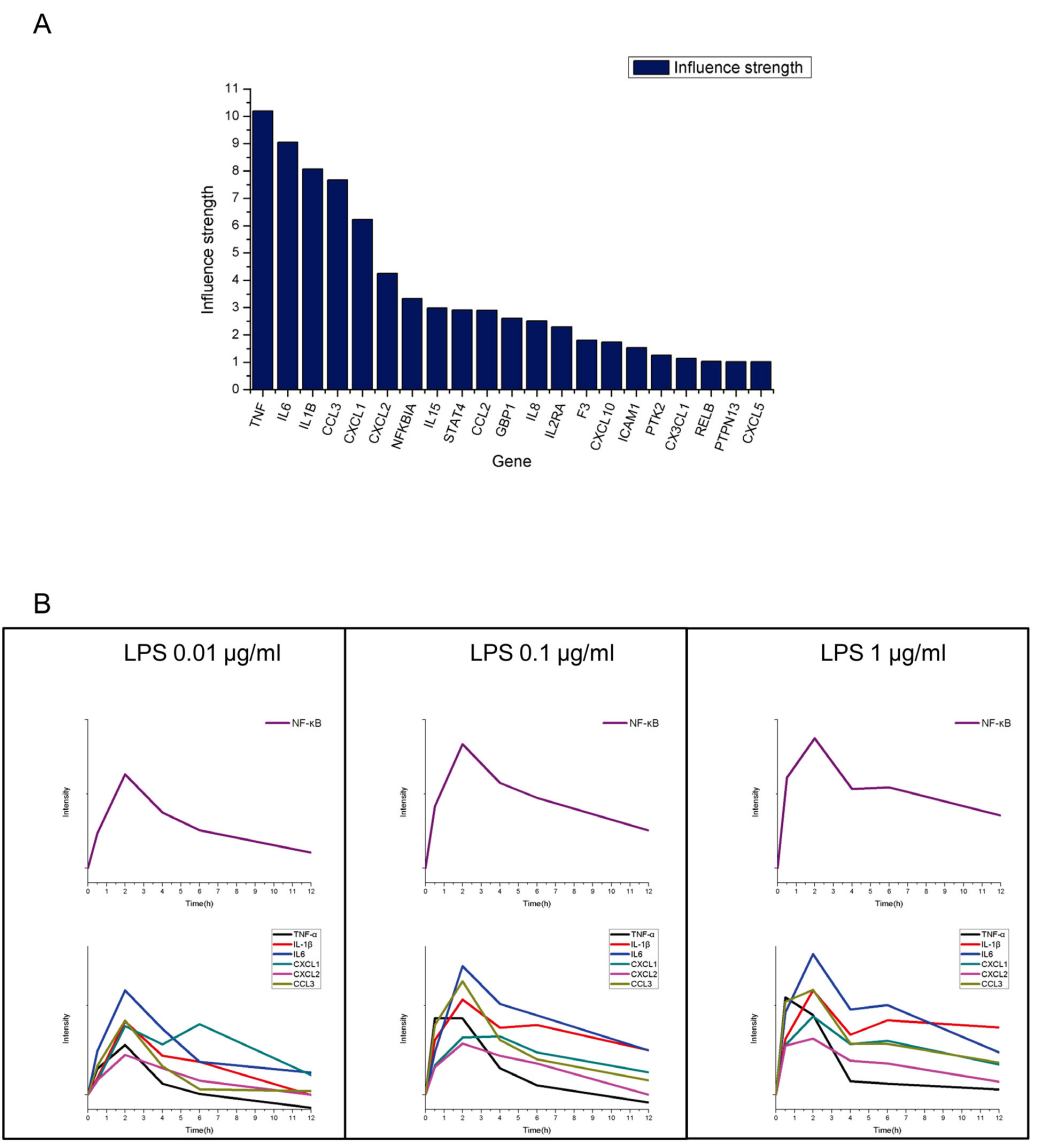
**The influence strength and the critical cytokines and chemokines**. (A) The top 20 influence strengths between the NF-κB activity and the regulated gene expression levels, which were derived from NCA. (B) There are six critical genes in our model because their gene expression levels are highly correlated with NF-κB activity. Most of them show sustained (non-decreasing) activity in the period between 4^th ^and 6^th ^hour in the highest dose of stimulus with 1 μg/ml LPS (right column).

In addition, Figure 7A illustrates the influence strengths of the NF-κB target genes. Strong influence strength means that the activity of the transcription factor and the target gene expression are highly correlated. The top six genes include, besides TNF-α and IL-1, other cytokines and chemokines, namely IL-6, CXCL1, CXCL2, and CCL3. All of these genes exhibit high correlations with the NF-κB profiling (Figure 7B). Interestingly, most of them have a second peak in the highest dose of stimulus with 1 μg/ml LPS. The Pearson correlation coefficients between them and NF-κB ranges from 0.78 to 0.96. Thus, the influence strength can be seen as the degree of the efficacy of a transcription factor to activate or inhibit one gene.

TNF-α and IL-1 are the main pro-inflammatory mediators of inflammation and tissue damage that are induced by NF-κB and associated with septic shock in acute severe infection. They play a synergistic role in orchestrating the inflammatory response and enhance the NF-κB activities by positive feedback loops. This positive effect of increasing TNFα and IL-1 on the NF-κB activity might explain the second wave of the NF-κB profile predicted by our integrative model in the presence of the highest LPS dose. Large amounts of TNF-α and IL-1 would result in a systemic inflammatory response syndrome (SIRS). This phenomenon leads to increased vascular permeability and the generation of thrombi in many small vessels; it also consumes massive amounts of blood-clotting proteins, thus deregulating hemostasis in the patient. The phenomenon also frequently results in the failure of several organs in the body; thus, septic shock often results in a high mortality rate [58,59].

IL-6 is also one of the most important mediators of the acute inflammatory response that is released in response to IL-1. IL-6 is responsible for the induction of fever; it stimulates energy mobilization, which then leads to an increased body temperature in the muscle and fatty tissue [60,61]. Moreover, in acute injury, high-dose bacterial invasion would induce acute inflammatory responses. The aberrant secretion of pro-inflammatory cytokines alters fibrin deposition and degradation and results in the abnormal cell-cell adhesion formation. CXCL1, a cytokine-induced neutrophil chemoattractant, has been revealed to be involved in cell-cell adhesion formation in sepsis [62], which is a serious challenge in acute infections and leads to high morbidity.

CXCL2 is also a critical chemokine for neutrophils. CXCL2 has been implicated in responses to both TNF-α and LPS via the NF-κB-dependent pathway. During infection or sepsis, increased CXCL2 would recruit mucosal neutrophils and amplify the inflammatory response. This might give rise to intestinal injury [63]. Besides the cytokines and chemokines described above, the expression of CCL3 also correlates with NF-κB activity, and NF-κB also strongly influences CCL3 expression. CCL3, a β-chemokine, regulates the migration and recruitment of various effector cells, such as monocytes, neutrophils, and natural killer cells, into infected sites. Previous studies provide evidence that CCL3 is involved in the degranulation and recruitment of mast cells [64]. The key roles of mast cells in the innate immunity against acute inflammation include the release of TNF-α-histamine and other inflammatory mediators, as well as bacterial phagocytosis. CCL3 can activate neutrophils and enhance the phagocytic antibacterial functions of macrophages. CCL3, therefore, is considered to play a crucial role in mast cell-associated host resistance against sepsis or other acute inflammation.

## Conclusions

In summary, we have linked a signaling pathway with the resulting gene responses by mapping the transcription factor activity (e.g., NF-κB) inferred from forward and reverse engineering for three different stimuli strengths. The corresponding signal profiles (e.g., IKK) were successfully reconstructed by our proposed method. Furthermore, the genes that highly correlated with transcription factor activity and had relatively strong influence strengths were also identified to reflect the biological consequences. This method is a practical and effective solution for linking exogenous cellular stimuli with signal transduction dynamics and gene expression profiling. It provides an overall systematic view of the inflammation process. In the near future, this strategy may be applied to more systems, as genome-wide regulatory data (such as ChIP-chip, ChIP-PET) involving transcription factors in mammals become available. For example, some inflammatory and autoimmune diseases are caused by the aberrant regulation of NF-κB, e.g., rheumatoid arthritis, inflammatory bowel disease, multiple sclerosis, atherosclerosis, and asthma [65,66]. Therefore, the application of our approach, which utilizes the dynamic simulation of a signaling pathway and reverse engineering, will be beneficial in the development of an in silico model for drug design and effective therapeutic strategies.

## Methods

### Network component analysis

In this work, a physical approach, the NCA proposed by Liao et al. [33], was used to reconstruct a model of the activity of transcription factors and the regulation strength from gene expression profiles [41]. The relationship between the activity of transcription factors and gene expression can be modeled by a log-linear combination. The gene expression level at each time point can be described as:

where *e*_*ij *_is the expression level of gene *i *at the *j^th ^*time point, *A*_*kj *_is the activity intensity of transcription factor *k *at the *j^th ^*time point and *s*_*ik *_is the influence strength of transcription factor *k *and gene *i*. Then we took the log of this equation, and the gene regulatory network is modeled as matrix representation:

The matrix [*E*]_*I *× *J *_is the logarithm of the signals of the microarray data containing *I *genes and *J *time points. [*S*]_*I *× *K *_represents the regulatory relationship and influence strength between *K *transcription factors and *I *regulated genes. [*A*]_*K *× *I *_represents the activities of the *K *transcription factors at the *J *time points.

Given [*E*]_*I *× *J*_, [*S*]_*I *× *K *_and [*A*]_*K *× *J *_can be obtained using a bipartite decomposition technique that requires knowledge of the position of the nonzero elements of [*S*]_*I *× *K *_, i.e., the prior knowledge of the regulatory relationships between the transcription factors and genes. The regulatory information was obtained from the TRANSFAC database (version 11.2) and MetaCore™ analytical suite, as shown in Figure 2. All of the NF-κB/RelA:P50-regulated genes in our network were expected to be significantly upregulated or downregulated.

### Forward simulation by HFPN

HFPN is an extension of Petri Net that can handle continuous events of real numbers [53]. It has the advantage of an intuitive representation for modeling a dynamic biological system. There are two kinds of places and transition components in HFPN, discrete/continuous places and discrete/continuous transitions. Discrete places and transitions hold nonnegative integer number tokens as their content. A continuous place can contain nonnegative real numbers. A continuous transition fires continuously according to a given speed function and values in the places. In addition, three kinds of arcs are defined. A normal arc makes the tokens in a place increase or decrease and triggers the next transition. An inhibitory arc is used to stop the action of transitions, and a test arc does not decrease tokens but triggers a connected transition.

In this study, the NF-κB signaling model was implemented by HFPN using the revised ODE model proposed by Hoffmann and colleagues [45,51,52]. This ODE model was modified from an earlier version [67] to include IKK time-course generator, nuclear-cytoplasmic volume ratio and other important factors as suggested by Lipniacki et al [68].

We created building blocks of HFPN according to a reaction categorized by the NF-κB signaling model, as shown in Figure S1a (Additional file 2). They include association, dissociation, nuclear import/export, and protein/RNA synthesis and degradation. The proteins are represented by continuous places, and the reactions are represented by continuous transitions. Based on these building blocks, the NF-κB signaling model was implemented in Cell Illustrator 3.0 [69] as shown in Figure S1b (Additional file 2).

We obtained NF-κB profiles by using different IKK profiles in this simulation model. To map the NF-κB profile from gene expression data, we developed an IKK generator, which generated the possible IKK profiles that had different peak times and intensities by adjusting the slope between each time point. The NF-κB profile was mapped in the order of time points at 0.5 h, 2 h, 4 h, 6 h, and 12 h.

### Normalizing and mapping the pattern of transcription factor profiles

Based on the simulation model of the IKK-NF-κB signaling pathway, we generated IKK activity curves by tuning the various slopes between each time point as the simulation input. To map the pattern of transcription factor profiles obtained from the signaling simulation to those obtained using NCA, we normalized these profiles to the same scale of intensity. The transcription factor profiles that were derived from the NCA represented the relative intensity among all time points. Thus, these profiles were normalized to have a maximum intensity of 1.0 by the following equation:

 is transcription factor activity obtained by the NCA at the *j^th ^*time point and  is the relative transcription factor activity. On the other hand, we also normalized the transcription factor concentration profiles from the signal transduction simulation using the same method. However, the log of the value obtained was used as the transcriptional activity because gene expression data used in the NCA were recorded as a log ratio. The relative transcriptional activity is given by:

After the transcription factor profiles from the NCA and signal simulation were normalized, we mapped them by calculating the Pearson correlation between the  obtained using different IKK stimuli profiles and the  reconstructed from the gene expression to determine which IKK stimulus profile could induce the gene response. Further, the actual activity profile of a transcription factor can be obtained from the signaling simulation, and the connectivity strength can be derived from the NCA equation using the actual activity profile of the transcription factor.

### IKK Kinase Assay

The stimulation was performed with the application of LPS at 1, 0.1, and 0.01 μg/ml to the THP-1 human monocytic cell line (American Type Culture Collection), which is used as a cell model system for PBMC [70]. At the indicated time points, before infection and at 0.5, 2, 4, 6, and 12 hours after infection, cells were harvested from the dish, washed twice by PBS, pelleted at 500 × g for 3 min, and snap-frozen in liquid nitrogen. The cell pellet was resuspended in lysis buffer containing 50 mM Tris-HCl, pH 7.6, 250 mM NaCl, 3 mM EDTA, 3 mM EGTA, 1% (v/v) Triton X-100, 0.5% (v/v) Igepal CA-630, 10% glycerol, 20 mM NaF, 40 mM β-glycero-3-phosphate, 2 mM DTT, 1 mM PMSF, 2 mM pNPP, 1 mM Na_3_VO_4_, and 10 μg/mL each of aprotinin, bestatin, leupeptin, and pepstatin.

The immuno-precipitation of the cell extract was performed using an anti-IKKγ Ab (BD). Protein A agarose beads were added to pull down the immuno-precipitated IKK complex. The immuno-pellets were washed and resuspended in 20 μl of kinase buffer containing 200 mM HEPES, pH 7.5, 100 mM MgCl_2_, 5 μCi of [γ-32P]-ATP and 1 μg of GST-IκBα(1-54) substrate and incubated for 30 min at 30°C. The reactions were stopped by the addition of SDS-PAGE loading buffer, heated at 95°C for 5 minutes, and resolved on 10% SDS-PAGE gels by standard procedures. The bottom section of the gel containing the phosphorylated GST-IκBα(1-54) as mounted, and the isotope intensity was detected by film. Proteins in upper section of the gel were transferred onto PVDF membrane (Millipore) and subjected to immunoblotting techniques to detect IKKα (Santa Cruz). The data were analysis by Image Q (GE Healthcare).

## Abbreviations

NCA: network component analysis; TNF-α: tumor necrosis factor alpha; IL-1: interleukin-1; LPS: lipopolysaccharide; HFPN: Hybrid Functional Petri-Net; PCC: Pearson correlation coefficient.

## Authors' contributions

SCP developed the method, performed the analyses and wrote the manuscript. DSHW advised on method design and wrote the manuscript. KCT and CCC performed the experiments, YYC and CHP performed the data analyses. YJC designed the experiments, interpreted the data and wrote the manuscript. CYT investigated the principle.

## Supplementary Material

Additional file 1 **Activity and strength matrices**. This file contains the activity matrices and strength matrices determined by NCA. Click here for file

Additional file 2 **The simulation model of the NF-κB signaling pathway constructed by Cell Illustrator**. This file contains Supplementary Figure S1 showing the simulation model of the NF-κB signaling pathway constructed by Cell Illustrator. The simulation model of the NF-κB signaling pathway was proposed by Hoffman et al. [45,51,52]. This model contains 24 components and 72 reactions and was rebuilt based on the Hybrid Functional Petri Net (HFPN) in Cell Illustrator 3.0. (A) The basic building blocks of HFPN according to the reaction category of the NF-κB signaling model. (B) The full model of the NF-κB signaling pathway that can yield the NF-κB profile using the input IKK profile. Click here for file
