# Supplementary material for: Computational modeling with forward and reverse engineering links signaling network and genomic regulatory responses: NF-κB signaling-induced gene expression responses in inflammation
Source: BMC Bioinformatics. 2010 Jun 8;11:308. doi: 10.1186/1471-2105-11-308 (PMC2889938; doi:10.1186/1471-2105-11-308)
Supplement: Additional file 2 — The simulation model of the NF-κB signaling pathway constructed by Cell Illustrator. This file contains Supplementary Figure S1 showing the simulation model of the NF-κB signaling pathway constructed by Cell Illustrator. The simulation model of the NF-κB signaling pathway was proposed by Hoffman et al. [45,51,52]. This model contains 24 components and 72 reactions and was rebuilt based on the Hybrid Functional Petri Net (HFPN) in Cell Illustrator 3.0. (A) The basic building blocks of HFPN according to the reaction category of the NF-κB signaling model. (B) The full model of the NF-κB signaling pathway that can yield the NF-κB profile using the input IKK profile. [file 1471-2105-11-308-S2.PDF]

Figure S1

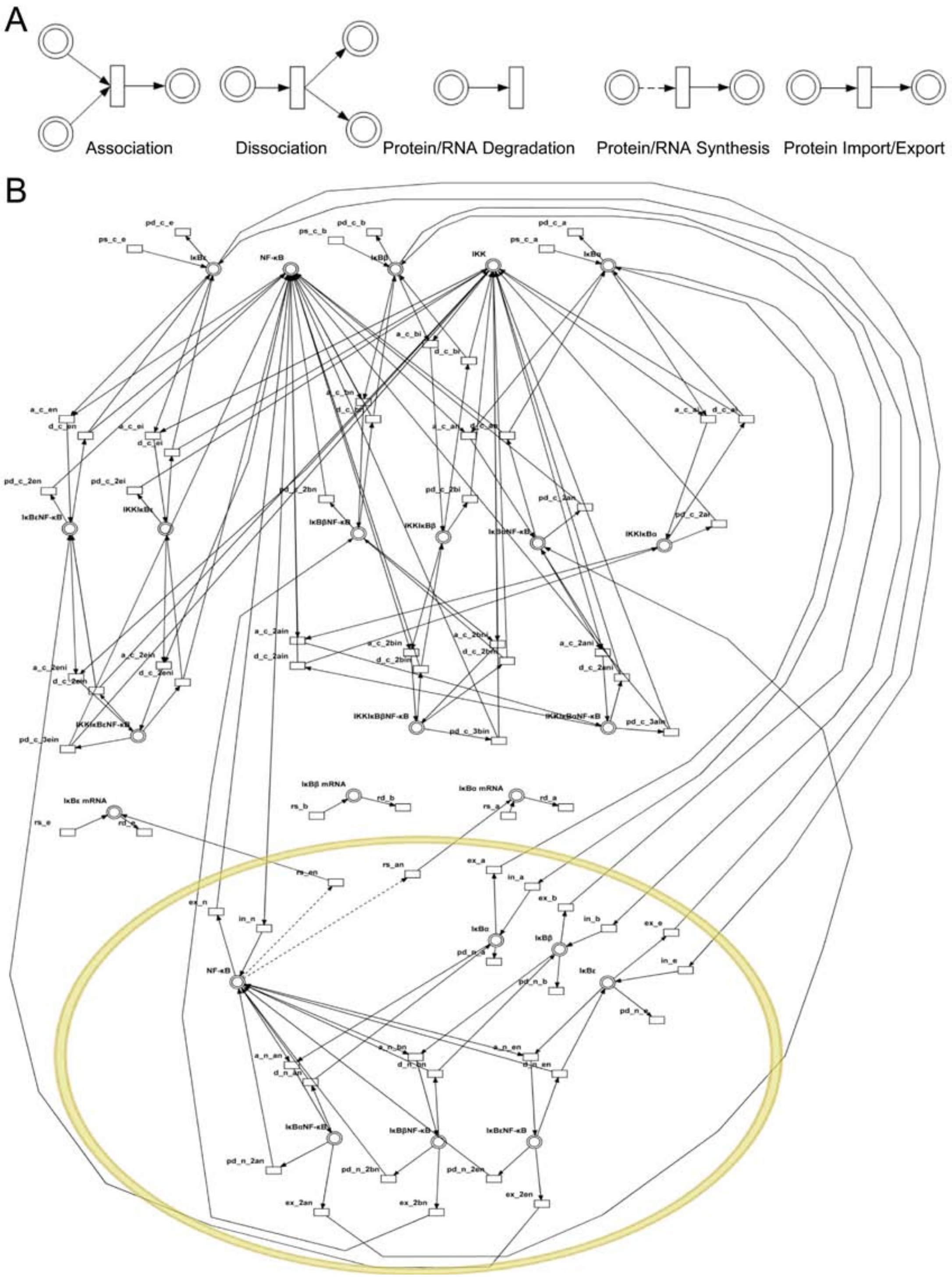

Figure S1. The simulation model of the NF-κB signaling pathway constructed by *Cell Illustrator*.

The simulation model of the NF-κB signaling pathway was proposed by Hoffman et al.[45], [51], [52]. This model contains 24 components and 72 reactions, and we rebuilt it as a Hybrid Functional Petri Net (HFPN) in Cell Illustrator 3.0. (A) The basic building blocks of the HFPN according to the reaction category of the NF-κB signaling model. (B) The full model of the NF-κB signaling pathway, which can

obtain the NF-κB profile by inputting IKK profile.
